# H-NST Induces LEE Expression and the Formation of Attaching and Effacing Lesions in Enterohemorrhagic *Escherichia coli*

**DOI:** 10.1371/journal.pone.0086618

**Published:** 2014-01-21

**Authors:** Jonathan A. Levine, Anne-Marie Hansen, Jane M. Michalski, Tracy H. Hazen, David A. Rasko, James B. Kaper

**Affiliations:** 1 Department of Microbiology and Immunology, University of Maryland School of Medicine, Baltimore, Maryland, United States of America; 2 Graduate Program in Biochemistry and Molecular Biology, University of Maryland, Baltimore, Maryland, United States of America; 3 Institute for Genome Sciences, University of Maryland School of Medicine, Baltimore, Maryland, United States of America; The Ohio State University, United States of America

## Abstract

**Background:**

Enteropathogenic *E. coli* (EPEC) and enterohemorrhagic *E. coli* are important causes of morbidity and mortality worldwide. These enteric pathogens contain a type III secretion system (T3SS) responsible for the attaching and effacing (A/E) lesion phenotype. The T3SS is encoded by the locus of enterocyte effacement (LEE) pathogenicity island. The H-NS-mediated repression of *LEE* expression is counteracted by Ler, the major activator of virulence gene expression in A/E pathogens. A regulator present in EPEC, H-NST, positively affects expression of H-NS regulon members in *E. coli* K-12, although the effect of H-NST on *LEE* expression and virulence of A/E pathogens has yet-to-be determined.

**Results:**

We examine the effect of H-NST on *LEE* expression and A/E lesion formation on intestinal epithelial cells. We find that H-NST positively affects the levels of *LEE*-encoded proteins independently of *ler* and induces A/E lesion formation. We demonstrate H-NST binding to regulatory regions of *LEE1* and *LEE3*, the first report of DNA-binding by H-NST. We characterize H-NST mutants substituted at conserved residues including Ala16 and residues Arg60 and Arg63, which are part of a potential DNA-binding domain. The single mutants A16V, A16L, R60Q and the double mutant R60Q/R63Q exhibit a decreased effect on *LEE* expression and A/E lesion formation. DNA mobility shift assays reveal that these residues are important for H-NST to bind regulatory *LEE* DNA targets. H-NST positively affects Ler binding to *LEE* DNA in the presence of H-NS, and thereby potentially helps Ler displace H-NS bound to DNA.

**Conclusions:**

H-NST induces *LEE* expression and A/E lesion formation likely by counteracting H-NS-mediated repression. We demonstrate that H-NST binds to DNA and identify arginine residues that are functionally important for DNA-binding. Our study suggests that H-NST provides an additional means for A/E pathogens to alleviate repression of virulence gene expression by H-NS to promote virulence capabilities.

## Introduction

The histone-like nucleoid structuring protein (H-NS) of *Escherichia coli* is the prototype of an important family of regulatory proteins that repress transcription of numerous genes in Gram-negative bacteria [1], [2]. H-NS helps bacteria respond to a wide range of environmental conditions such as changes in pH, osmolality and temperature [3]. In *E. coli* K-12, H-NS is a small, 15.9 kDa protein composed of 137 amino acids. H-NS-mediated modulation of gene expression can involve multiple mechanisms including binding of H-NS to regulatory regions of H-NS regulon genes to block association of RNA polymerase or by preventing open-complex formation after RNAP has already associated with the promoter [1], [4]–[6]. These mechanisms can be augmented or countered by other nucleoid-associated proteins such as Hha, YmoA, Fis, HU, and IHF [1], [6]. The N-terminal coiled-coil region of H-NS functions in oligomerization, either forming multiple homo-oligomeric states or heteromers with H-NS paralogs such as StpA, and Hha/YmoA family of proteins [1], [4], [6]. The C-terminal region of H-NS is the DNA-binding domain. The H-NS family of proteins contains a conserved DNA-binding motif that shares preferences for curved AT-rich DNA targets [3], [7], [8].

In addition to modulating expression of backbone chromosomal genes in *E. coli* K-12 such as *proU* and *bgl* [3], [9], H-NS also plays a key role in regulating virulence factors of many bacterial pathogens, including *Shigella*, *Salmonella*, enteropathogenic *E. coli* (EPEC) and enterohemorrhagic *E. coli* (EHEC) [1]. The majority of genes encoding virulence factors in these pathogens are contained in pathogenicity islands or other mobile genetic elements, which are AT-rich compared to chromosomal housekeeping genes. These DNA sequences thought to be obtained via lateral gene transfer are termed xenogenic (i.e., derived from a foreign source) [6]. Repression of such genes would presumably provide an evolutionary advantage in allowing these genes to be less likely to have a deleterious effect than if they were unregulated. H-NS, while encoded in the chromosomal backbone of these pathogens, can interact with other regulatory proteins encoded by pathogenicity islands to modulate virulence gene expression that allows pathogens to adapt to the host environment.

**Figure 1:**
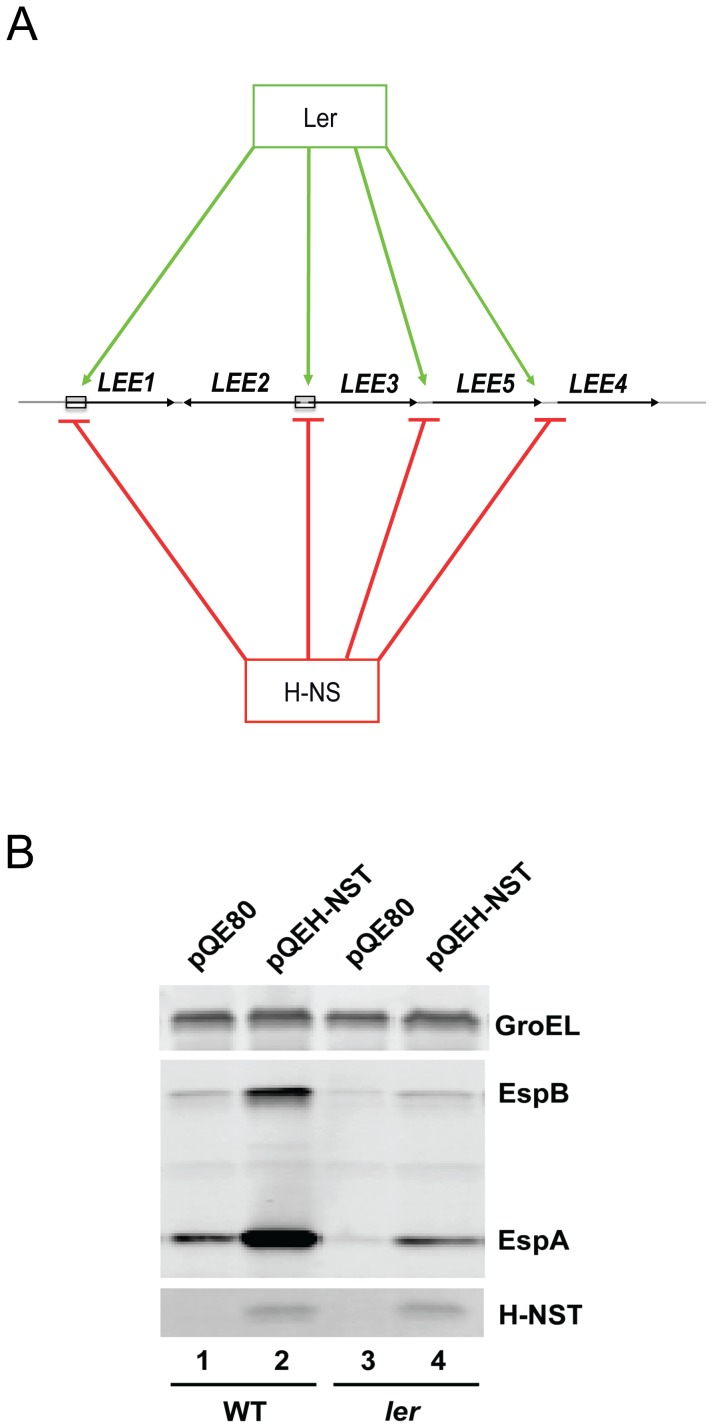
H-NST positively affects *LEE*-encoded protein levels. (A) Regulation of the five major *LEE* operons, *LEE1-5*, by H-NS (red) and Ler (green). Positive regulation by Ler and repression by H-NS are indicated by open and blocked arrow heads respectively. Boxes indicate the approximate locations of the regulatory regions of *LEE1* and *LEE2/3* investigated in this study. (B) The effect of EPEC H-NST on the levels of *LEE*-encoded proteins in EHEC was determined by western analysis as described in *Materials and Methods*. Wild type EHEC TUV93-0 (lanes 1 and 2) and a *ler-*deleted derivative (lanes 3 and 4) containing the empty expression vector pQE80 (lanes 1 and 3) or pQE80H-NST (lanes 2 and 4) were grown in LB to a density of OD_600_∼0.5 followed by induction with 0.5 mM IPTG for 30 min. The *LEE*-encoded T3SS proteins EspA and EspB were detected by western analysis of total protein using polyclonal antisera against the respective proteins as indicated. His-tagged H-NST was detected using a tetra-His antisera. GroEL served as a loading control for total protein. Data shown are representative of four independent experiments.

One group of gastrointestinal pathogens that illustrates this interaction of H-NS and pathogenicity island-encoded regulators is the attaching and effacing (A/E) pathogens [10], named for the pathognomonic intestinal histopathology characterized by intimate adherence of the bacteria to epithelial cells and effacement of microvilli. EPEC causes diarrhea, primarily in infants, while EHEC causes bloody diarrhea and the potentially fatal hemolytic uremic syndrome. In addition to these human pathogens, there are also A/E pathogens for rabbits (rabbit EPEC or REPEC) and for mice (*Citrobacter rodentium*) [11]. All of these pathogens contain the horizontally-acquired Locus of Enterocyte Effacement (LEE) pathogenicity island, which is primarily responsible for the A/E histopathology [12]–[19]. The LEE pathogenicity island contains 41 genes with the majority located in the five operons *LEE1-5* [20]–[23] (figure 1A). The majority of *LEE* genes encode a type III secretion system (T3SS) that resembles a needle-like structure, with the EspA protein forming the filament and EspB/EspD forming the pore inserted into the host cell. Effector proteins are secreted through the needle-like structure into the host cell where they manipulate host signaling pathways to subsequently induce disease [24], [25]. Deletion of the *hns* gene encoding H-NS greatly increases transcription of many *LEE* genes [12], [16], [26].

The first gene in the *LEE1* operon encodes the *LEE*-encoded regulator (Ler), an H-NS-like protein that shares 36% amino acid sequence identity to the DNA-binding C-terminal domain of H-NS. Ler, composed of 123 amino acids (14.3 kDa), is the master positive regulator of EPEC and EHEC *LEE* virulence genes such as *espA and espB*, as well as non-*LEE*-encoded virulence factors such as the long polar fimbria (*lpf1*) and a serine protease (*stcE*) [27]–[30]. As a positive regulator of virulence gene expression, Ler counteracts H-NS-mediated repression, probably by binding to DNA and displacing H-NS from regulatory regions of the Ler regulon [27], [31], [32]. Oligomerization of Ler, like H-NS, occurs through the coiled-coil region located in the N-terminus [30], [31]. DNA-binding activity of Ler involves the C-terminus, in particular the Arg90 residue lodged in the conserved DNA-binding motif of the H-NS family of proteins [1], [6], [25], [30], [33]–[35]. Ler preferentially binds to curved AT-rich DNA including a 10 bp long DNA sequence of the *LEE2/LEE3* regulatory region, which was identified as a binding target for the Ler C-terminal DNA-binding domain [30], [36]. The specific binding to the *LEE2/LEE3* target DNA involves the side chain of Arg90 being inserted into a narrow minor groove while Arg93 helps in stabilizing the DNA protein complex [36]. Both oligomerization and DNA binding are essential for Ler antagonism of the H-NS repression. Antagonizing H-NS repression leading to increased gene expression is not exclusive to Ler, since other H-NS-modulating proteins have this effect by a different mechanism of forming dominant-negative oligomers [1], [2], [6], [37]–[39].

Inhibition of H-NS activity is also seen with the gene *5.5*-encoded protein of T7 phage (gp 5.5) and the H-NS truncated protein (H-NST) of EPEC, both of which have been shown to interact with H-NS and hinder its repressive activity [37], [39]–[42]. In *E. coli* K-12 the gp 5.5 protein has been shown to relieve H-NS-mediated repression of the *proU* promoter *in vivo* [38], and was shown to diminish H-NS binding to the *bglG* promoter region via interaction with the H-NS oligomerization domain, forming a dominant-negative oligomer [39]. Although DNA-binding activity has yet-to-be demonstrated for gp5.5, it was reported to form a complex with H-NS and tRNA to mask tRNA priming in T7 DNA replication [40]. The 80-residue (10.5 kDa) protein H-NST is not present in *E. coli* K-12 or EHEC but is encoded in the chromosome of some isolates such as EPEC E2348/69, uropathogenic *E. coli* (UPEC) strain CFT073 and *C. rodentium.* H-NST from EPEC is encoded by a pathogenicity island located at the *asnW* locus. Though H-NST exhibits an overall low amino acid sequence similarity to H-NS of only 29%, the first 43 residues of H-NST share 40% similarity to H-NS [37]. Williamson *et al* demonstrated that EPEC H-NST negatively affects H-NS-mediated repression of the *E. coli* housekeeping genes *proU* and *bgl* by forming dominant-negative hetero-oligomers with H-NS that render H-NS inactive when tested in a *E. coli* K-12 background. These authors further demonstrated that Ala16 of H-NST is important for oligomerization and thereby activity [37]. DNA-binding activity of H-NST has not been demonstrated nor has relief of H-NS-mediated repression by H-NST yet been shown for virulence factor genes in pathogenic *E. coli*.

In this study, we assess the effect of H-NST on H-NS-mediated regulation of *LEE* expression. We show that H-NST positively affects levels of *LEE*-encoded proteins and A/E lesion formation. We demonstrate that H-NST specifically binds to *LEE* regulatory DNA regions and further show that Ala16 is required for H-NST-mediated increase in the levels of *LEE*-encoded proteins and induction of A/E lesion formation. Additionally, we determine H-NST Arg60 and Arg63 residues to be important for the ability of H-NST to bind DNA, resulting in the induction of *LEE* expression and A/E lesion formation. Further, we demonstrate that H-NST is conserved among many human and plant bacterial pathogens, suggesting a global role of H-NST in regulating the expression of the H-NS regulon.

## Materials and Methods

### Standard procedures

Standard DNA techniques, liquid media and agar plates were used as described [43]. Restriction endonucleases, T4 DNA kinase- and ligase were used as recommended by the manufacturer (New England Biolabs). DNA used for cloning purposes was PCR amplified using the high-fidelity DNA polymerases Phusion Flash (Fermentas) or Easy-A (Agilent). DNA oligonucleotides were obtained from Intergrated DNA Technologies and DNA sequencing was performed by the University of Maryland Biopolymer-Genomics Core Facility. Bacteria were grown at 37°C in LB or DMEM (Invitrogen #11885) media supplemented with ampicillin (100 µg/ml) (Sigma) as needed. HeLa cells (ATTC #CCL-2) were cultured in DMEM/F12 (Invitrogen #11330) supplemented with 10% fetal bovine serum (FBS), 100 U/ml penicillin and 100 µg/ml streptomycin (Invitrogen) at 37°C in 7% CO_2_.

### Plasmid constructions

Oligonucleotide sequences used for plasmid constructions are listed in table 1.

**Table 1:** Oligonucleotides used in this study.

*Application*/name	Oligonucleotide sequence (5′ to 3′)
*Plasmid construction*	
QEH-NST F	AGCAGGATCCATGATTGATGAATTTCATGTGATGTATATGTAT
QEH-NST R	CACCGAAGCTTCAGTCAATGAGATCTTCTGGCGAAAC
QEH-NS F	AGCAGGATCCATGAGCGAAGCACTTAAAATTCTGAACAAC
QEH-NS R	CACCGAAGCTTATTGCTTGATCAGGAAATCGTCGAG
*Site-directed mutagenesis*	
H-NSTA16V F	TATATGTATAAAAAAATCCAAGTAGAAGCCGCAACCACTGACCTC
H-NSTA16V R	GAGGTCAGTGGTTGCGGCTTCTACTTGGATTTTTTTATACATATA
H-NSTA16L F	TATATGTATAAAAAAATCCAAGCAGAAGCCGCAACCACTGACCTC
H-NSTA16L R	GAGGTCAGTGGTTGCGGCTTCTGCTTGGATTTTTTTATACATATA
H-NSTR60Q F	CGTAAGTTGAAAATGAAACAAGCACAAAGATTACTTGAG
H-NSTR60Q R	CTCAAGTAATCTTTGTGCTTGTTTCATTTTCAACTTACG
H-NSTR60Q/R63Q F	TTGAAAATGAAACAAGCACAACAATTACTTGAGAAAATGGCATGTGACCGGG
H-NSTR60Q/R63Q R	CCCGGTCACATGCCATTTTCTCAAGTAATTGTTGTGCTTGTTTCATTTTCAA
*EMSA DNA fragments*	
LEE1P2 F	TTAAGGTGGTTGTTTGATGA
LEE1P2 R	TTTGGATTCAGCAAA
LEE1P1P2 F	GCAATGAGATCTATCTTATAAAGAGAAACGC
LEE3 F	GTTGAAGAGTTTTTAAGATTGTTGG
LEE3 R	ATAAATAATCTCCGCATGCT
rssB F	TGCAAGTCGAACGGTAACAG
rssB R	AGTTATCCCCCTCCATCAGG

pQEH-NST: A 262 bp DNA fragment encoding *hnsT* was PCR amplified from EPEC E2348/69 gDNA using the primer set QEH-NST F/QEH-NSTF R, digested with *Bam*HI and *Hind*III and cloned into the corresponding sites in pQE80 (QIAGEN). Plasmid pQEH-NST encodes a recombinant C-terminal His-tagged H-NST.

pQEH-NS: A 434 bp DNA fragment encoding *hns* was PCR amplified from EPEC E2348/69 gDNA using primer set QEH-NS F/QEH-NS R, digested with *Bam*HI and *Hind*III and cloned into the corresponding sites in pQE80 (QIAGEN). Plasmid pQEH-NS encodes a recombinant C-terminal His-tagged H-NS.

H-NST mutant derivatives were constructed by site-directed mutagenesis of pQEH-NST using the QuickChange XL Site-directed Mutagenesis Kit (Agilent) according to manufacturer's instructions. The plasmids pQEH-NST A16V, pQEH-NST A16L, pQEH-NST R60Q and pQEH-NST R60Q/R63Q encoding H-NST substitution mutants were generated using the primer sets H-NSTA16V F/H-NSTA16V R, H-NSTA16L F/H-NSTA16L R, H-NSTR60Q F/H-NSTR60Q R and H-NSTR60Q/R63Q F/H-NSTR60Q/R63Q R respectively.

### BLAST searches and multiple amino acid sequence alignment

BLAST searches were used to identify H-NST present among non-redundant protein sequences in the NCBI database using the BLASTp program with an expect threshold of 10 and the scoring parameters: Blosum 62 matrix, gap cost was 11 for existence and 1 for extension, and conditional compositional score matrix adjustment (www.ncbi.nlm.nih.gov). H-NST from EPEC strain E2348/69 (YP 002329609) was used as query sequence. Proteins identified at a threshold e-value of 2×10^−26^ or less with sequence coverage of at least 77% were considered. In addition, a BLASTn search of a database containing a collection of 114 A/E *E. coli* isolates and 24 reference strains [44] was performed using a threshold e-value of 1×10^−15^ to identify genes encoding *hnsT*. The multiple amino acid sequence alignment of H-NST was prepared using ClustalW2 [45], [46].

H-NST proteins from the following 65 strains were used to generate the multiple sequence alignment: *C. rodentium* ICC168 (YP003365612), *Dickeya zeae* (WP019843943), *Enterobacter* sp. SST3 (EJO48231), *E. coli* 113303 (ESA61347), *E. coli* 2362-75 (EFR16544), *E. coli* 2848050 (EMW14361), *E. coli* 3003 (EII86141), *E. coli* 536 (YP669831), *E. coli* C262-10 (AIAP01000001.1), *E. coli* C639-08 (AIBH01000001.1), *E. coli* C844-97 (AIBZ01000001.1), *E. coli* C93-11 (AICD01000002.1), *E. coli* CFT073 (NP754305), *E. coli* CUMT8 (EIL77087), *E. coli* DEC1A (EHU10545), *E. coli* DEC1B (EHU13728), *E. coli* DEC1C (EHU11552), *E. coli* DEC1D (EHU23639), *E. coli* DEC1E (EHU27188), *E. coli* DEC2A (EHU30556), *E. coli* DEC2B (EHU39793), *E. coli* DEC2C (EHU44940), *E. coli* DEC2D (EHU46227), *E. coli* DEC12A (EHX20918), *E. coli* DEC12E (EHX46652), *E. coli* DEC15B (EHX98907), *E. coli* DEC15C (EHX01733), *E. coli* DEC15D (EHX09544), *E. coli* DEC15E (EHX13659), *E. coli* E2348/69 (YP002329609), *E. coli* E851/71 (ALNX00000000), *E. coli* HVH 125 (4-2634716) (EQR42309), *E. coli* HVH HVH 225 (4-1273116) (EQV31905), *E. coli* HVH 154 (4-5636698) (EQS65207), *E. coli* HVH 158 (4-3224287) (EQS57084), *E. coli* KTE100 (EOW07218), *E. coli* KTE157 (ELJ10809), *E. coli* KTE16 (ELC28364), *E. coli* KTE192 (ELH08668), *E. coli* KTE227 (ELH91245), *E. coli* MS 21-1 (EFK17805), *E. coli* MS 57-2 (EGB75116), *E. coli* MS 115-5 (EFJ95479), *E. coli* MS 200-1 (EFJ59489), *E. coli* 042 (CBG35676), *E. coli* OK1357 (EFZ69143), *E. coli* TA124 (EHN89354), *E. coli* TW07793 (EII95637), *E. coli* UMEA 3022-1 (EQW12951), *E. coli* UMEA 3108-1 (EQW66083), *E. coli* UMEA 4076-1 (ERA50029), *Klebsiella oxytoca* 10-5245 (EHT00587), *K. pneumoniae* UCICRE 7 (ESM00810), *Pantoea ananatis* PA13 (YP005993219), *Pectobacterium atrosepticum* SCRI1043 (CAG74542), *Pectobacterium wasabiae* CFBP 3304 (WP005969703), *Salmonella enterica* subsp. arizonae serovar 62:z4,z23:- strain RSK2980 (YP001569976), *S. enterica* subsp. diarizonae serovar 60:r:e,n,x,z15 strain 01-0170 (ESJ14503), *S. enterica* subsp. enterica serovar Anatum str. ATCC BAA-1592 (ESJ09538), *S. enterica* enterica subsp. enterica serovar Hvittingfoss strain A4-620 (EHC52821), *S. enterica* subsp. enterica serovar Indiana strain ATCC 51959 (ESG993750, *S. enterica* subsp. enterica serovar Muenster str. 660 (ESB61545), *S. enterica* subsp. enterica serovar Nchanga strain CFSAN001091 (ESJ38946), *S. enterica* subsp. enterica serovar Sloterdijk str. ATCC 15791 (ESF40572), and *Yersinia rohdei* ATCC 43380 (WP004713599).

### Western blot analysis

The effect of expressing H-NST from pQEH-NST on the production of T3SS-associated proteins was determined in the EHEC O157:H7 EDL933 Δ*stx1*Δ*stx2* strain TUV93-0 [47] and AMH101 [48], which is TUV93-0 containing an in-frame deletion of *ler.* Wild type H-NST and H-NST mutants were produced from pQEH-NST, pQEH-NST A16V, pQEH-NSTA16L, pQEH-NSTR60Q and pQEH-NSTR60Q/R63Q in TUV93-0. Cultures were grown in LB containing ampicillin at 37°C to a density of OD_600_∼0.5 followed by induction of H-NST expression with 0.5 mM IPTG for 1h. Total cellular protein was precipitated with 5% (vol/vol) trichloric acid, washed with acetone, resuspended in 1× Next Gel sample loading buffer (Amresco). Proteins were resolved on a 4–20% Tris-HCl Criterion precast protein gels (BioRad) and transferred onto an Immobilon-FL polyvinylidene difluoride membrane (Millipore) using a Trans-Blot SD Semi-Dry Transfer Cell (BioRad). The membrane was blocked in Odyssey blocking buffer (Li-Cor Biosciences), exposed to polyclonal antibodies specific to EspA, EspB and GroEL (Sigma), and subsequently to Alexa Fluor 680-conjugated goat anti-rabbit (Invitrogen). A monoclonal Tetra-His anti-mouse antisera (QIAGEN) and Alexa Fluor 680-conjugated goat anti-mouse (Invitrogen) were used to detect His-tagged H-NST. Detection of GroEL served as a loading control for total protein. Proteins were visualized and quantified using an Odyssey Infrared Imaging System with application software version 3.0 (Li-Cor Biosciences) as recommended. The levels of *LEE*-encoded proteins were normalized to that of GroEL. The western analyses were carried out on four independent biological samples for each strain.

### Fluorescent actin staining assay

The effects of wild type H-NST and H-NST mutants on the ability of EHEC O157:H7 TUV93-0 to induce A/E lesion formation on HeLa cell monolayers was evaluated using the fluorescent actin staining assay (FAS) [49]. Wild type H-NST and the H-NST substitution mutants A16V, A16L, R60Q and R60Q/R63Q were produced from pQEH-NST, pQEH-NSTA16V, pQEH-NSTA16L, pQEH-NSTR60Q and pQEH-NSTR60Q/R63Q respectively. Bacterial strains carrying the vector pQE80 or plasmids encoding *hnsT* and its mutant derivatives were inoculated from freezer stocks into LB medium containing ampicillin and grown statically for about 18 h at 37°C. Cultures were then diluted 1∶3 in DMEM containing 0.2% mannose, ampicillin and 0.3 mM IPTG to induce expression of *hnsT*, and grown statically at 37°C in 7% CO_2_ for 1 h. Levels of wild type and mutant H-NST produced in the preinduced TUV93-0 strains were confirmed by western blot analysis. Semi-confluent HeLa cell monolayers grown on glass coverslips to ∼80% confluence were co-cultured with an initial number of ∼1×10^6^ bacteria in DMEM supplemented with 2% FBS. After 3 h of infection cells were washed with Hanks buffer (Invitrogen), fresh media was added, and cells were incubated for an additional hour. At 4 h post infection cell monolayers were washed once with Hanks buffer, and fixed in 4% formamide in 1× PBS. Coverslips were washed three times with 1× PBS, cells were permeabilized with 0.1% Triton X-100 in 1× PBS, and F-actin was stained using Alexa Fluor 488 phalloidin (Invitrogen) diluted 1∶50. Coverslips were mounted on slides using Prolong Gold Antifade Reagent (Invitrogen). FAS assays were independently conducted at least three times for each strain. Actin-stained cells were visualized using an AxioSkop microscope equipped with a 40× objective and images were captured with an AxioCam MR3 digital camera using AxioVision v. 4.8 software (Carl Zeiss MicroImaging Inc).

### Protein production and purification

Recombinant H-NS, H-NST, and H-NS mutant derivatives were produced from pQEH-NS, pQEH-NST, pQEH-NSTA16V, pQEH-NSTA16L, pQEH-NSTR60Q and pQEH-NSTR60Q/R63Q in BL21-DE3 (pLysS) (Novagen). Cells were grown in 1 L of LB medium at 37°C to an optical density of OD_600_ ∼0.5 prior to the induction with 0.5 mM IPTG. Proteins were produced for 1 h and cells were then harvested by centrifugation at 7,667 *g* for 20 min at 4°C. Cell pellets were suspended in binding buffer (50 mM NaH_2_PO_4,_ 300 mM NaCl, 10% glycerol, 40 mM imidazole, pH 8) to a final volume of 40 ml. Cells were lysed by two passages through a Microfluidics LV1 micro-fluidizer, and then the lysed cell suspension was centrifuged at 26,536* g* for 70 min at 4°C. The supernatant of the lysate was then filtered through a 0.2 µm pore size filter (Millipore) and applied to a HisTrap FF column (GE Healthcare) coupled to an ÄKTAprime plus system (GE Healthcare). After sample application the column was first washed with 30 ml of binding buffer at a flow rate of 2 ml/min and then washed with 50 ml of 6% elution buffer (50 mM NaH_2_PO_4,_ 300 mM NaCl, 10% glycerol, 500 mM imidazole, pH 8) in binding buffer. Protein was eluted with 100% elution buffer and eluates were buffer exchanged into storage buffer (50 mM NaH_2_PO_4_, 300 mM NaCl, 10% glycerol, pH 7.4) and concentrated using an Amicon Ultra Centrifugal Filter Device with a 3 kDa MW cut-off value (Millipore). Protein samples were analyzed by SDS-PAGE using a 4–20% Tris-HCl Criterion precast gel (BioRad), and visualization with GelCode Blue Stain Reagent (Thermo Scientific), a technique that provides nanogram-level detection. The purity of the H-NST preparation was about 95% as estimated from the stained gel.

Recombinant Ler-Myc-His protein was produced from pVS45 in DH5α [30]. Cultures were grown in LB medium at 37°C to a density of O.D_600_ ∼0.5 before the expression of *ler* was induced with 0.2% arabinose for 2 h. Cells were harvested by centrifugation at 7,667 *g* at 4°C for 20 min. Purification of Ler-Myc-His was as described for H-NST with the exception that 10% glycerol was omitted from all buffers. Purified Ler was buffer exchanged and concentrated using Amicon Ultra Centrifugal Filter Device with a 10 kDa MW cut-off value.

### Preparation of fluorescently-labeled DNA fragments and Electrophoretic Mobility Shift Assays (EMSA)

#### Preparation of fluorescently-labeled DNA fragments

Fluorescently-labeled oligonucleotides used for PCR amplification of DNA fragments containing *LEE* regulatory regions were prepared as described [50]. Briefly, 5′-amine-modified oligonucleotides (Integrated DNA Technologies) were resuspended in 300 µl of dH_2_O, chloroform extracted three times, ethanol precipitated in 125 µM NaCl, and then resuspended in 300 µl dH_2_O. The oligonucleotides (5 µg) were fluorescently labeled in 100 µl reactions using a ten-fold molar excess of Alexa Fluor® 790 Carboxylic Acid, Succinimidyl Ester, penta (triethylammonium) Salt (Life Technologies) in 100 mM Sodium Tetraborate (pH 8.5). Reactions were allowed to proceed overnight at room temperature in the dark. Labeled oligonucleotides were purified using G-25 spin columns (GE Healthcare) according to manufacturer's directions.

DNA fragments encoding *LEE1* P2, *LEE1* P1P2 and *LEE3* regulatory regions were PCR amplified from EHEC strain TUV93-0 gDNA using the fluorescently-labeled primer sets LEE1P2 F/LEE1P2 R, LEE1P1P2 F/LEE1P2 R and LEE3 F/LEE3 R respectively. The *LEE1* P2 and *LEE1* P1P2 DNA fragments contain the *LEE1* regulatory region ranging from positions −112 to +33 and −301 to +33 relative to the transcription initiation site for the proximal *LEE1* promoter respectively. The *LEE3* DNA fragment contains the sequence from +83 to +210 relative to the transcription initiation site for *LEE3*. The primer set rssB F/rssB R was used to amplify a 99 bp long unlabeled DNA fragment containing part of *rssB*, which served as nonspecific DNA target. Amplified DNA products were purified using G-50 spin columns (GE Healthcare) according to manufacturer's directions.

#### Electrophoretic mobility shift assay

Purified H-NS, Ler, H-NST and mutant H-NST derivatives (concentrations as indicated in the figure legends) were incubated for one minute prior to addition of 24 ng of fluorescently-labeled DNA fragment containing the *LEE1* P2, *LEE1* P1P2 or *LEE3* regulatory regions and then incubated for 20 min at 30°C in binding buffer (10 mM Tris-HCL, 1 mM EDTA, 50 mM KCl, 10% glycerol, 50 µg/mL, 1 mM DTT, pH 7.4). Unlabeled *LEE* DNA fragments added in 6-fold excess and unlabeled *rrsB* DNA fragment added in 12-fold excess served as specific and nonspecific competitor DNA, respectively. Binding reactions containing both H-NST and H-NS were preincubated for 10 min at room temperature to allow interaction between proteins before the DNA fragment was added. Further, binding experiments where Ler was added to reactions already containing H-NST and/or H-NS bound to DNA were first incubated for 10 min at room temperature, and then Ler was added followed by 20 min of incubation at 30°C. DNA fragments were separated by polyacrylamide gel electrophoresis (PAGE) using a 4–20% TBE Criterion precast gel run in 1× TBE at 52 amps for 60 min (BioRad). Fluorescently-labeled DNA fragments were visualized using an Odyssey Imaging System at 800 nm with application software version 3.0 (Li-Cor Biosciences). The EMSA analyses were carried out at least three times using proteins from at least two different protein purification preparations.

## Results

### H-NST induces *LEE*-encoded protein levels independently of Ler

While H-NST encoded by EPEC is known to positively affect the expression of H-NS-controlled housekeeping genes in *E. coli* K-12 [37], the effect of H-NST on the expression of the *LEE* and the virulence-associated A/E lesion phenotype remains to be elucidated. To evaluate the effect of H-NST on *LEE*-encoded protein levels, we cloned *hnsT* from EPEC under the control of an IPTG-inducible *tac* promoter in pQE80 and produced H-NST from this construct in EPEC strain E2348/69. Production of H-NST from pQEH-NST in EPEC grown to the exponential phase in DMEM did not affect levels of *LEE*-encoded proteins (unpublished data), which might be due to the possibility that an effect of H-NST is masked by a relatively high basal level of *LEE*-encoded proteins present in EPEC. Since the abundance of *LEE*-encoded proteins in EHEC is minimal in the exponential phase [48], we assessed the regulatory effect of H-NST on the LEE using the EHEC O157:H7 strain TUV93-0, a *stx*-deleted derivative of EDL933 that exhibits Ler and H-NS-mediated regulation of the *LEE* similar to EPEC. Interestingly, production of H-NST from pQEH-NST in EHEC grown in LB to exponential phase (OD_600_∼0.4) induced the expression of the *LEE* as reflected by increased levels of the T3SS-secreted proteins EspA and EspB by 5.4-fold and 3.1-fold, respectively, as opposed to the vector control (figure 1B, lane 2). Moreover, production of *C. rodentium* H-NST, the amino acid sequence of which is 79% similar to that of EPEC H-NST, also increased levels of EspA and EspB in EHEC (unpublished data), suggesting that the H-NST proteins from both these A/E pathogens affect *LEE* expression. These results strongly suggest that production of H-NST has a dominant-negative effect on the H-NS-mediated repression of *LEE* expression in the exponential growth phase. Since our data indicated that H-NST positively affects levels of *LEE*-encoded proteins, we further investigated whether the *LEE*-encoded global virulence gene regulator Ler was required for regulation by H-NST. To assess this we provided H-NST from pQEH-NST in a *ler*-deleted derivative of EHEC TUV93-0, and found that production of H-NST increased EspA and EspB levels by 1.9-fold and 1.2-fold respectively (figure 1B, lane 4), indicating that H-NST positively affects levels of EspA independently of *ler* probably by hindering H-NS activity as previously suggested [37].

### H-NST is conserved in various *Enterobacteriaceae* including A/E pathogens

**Figure 2:**
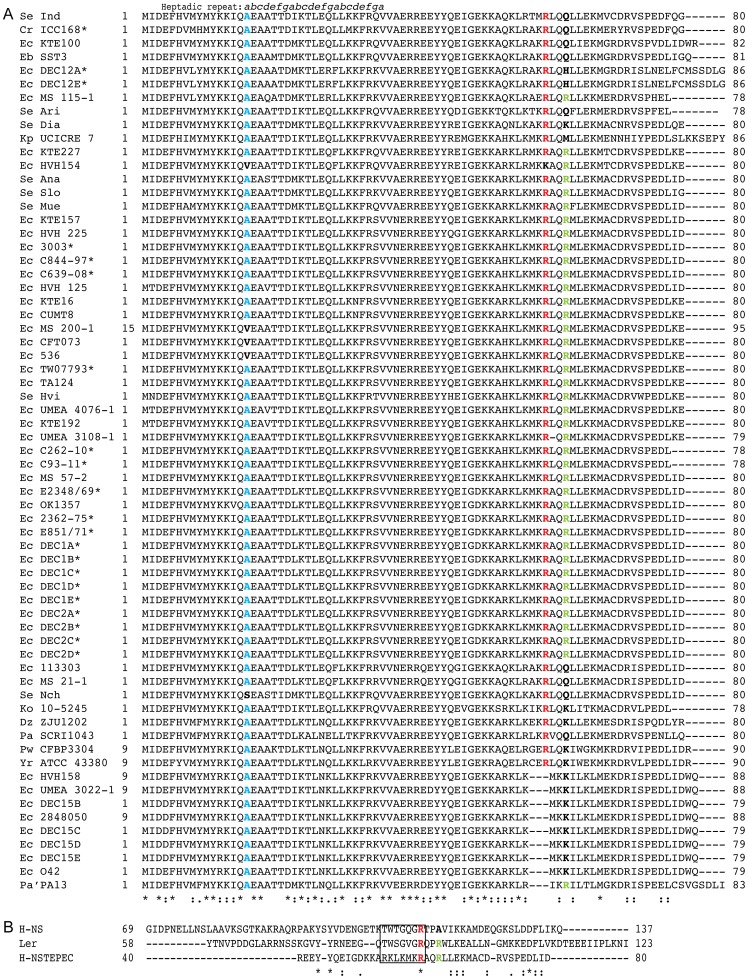
H-NST is conserved in various enteropathogens. (A) ClustalW2 sequence alignment of 65 H-NST homologs from 65 *Enterobacteriaceae*. The following strain abbreviations were used: Cr (*C. rodentium*), Dz (*Dickeya zeae*), Eb (*Enterobacter*), Ec (*E. coli*), Ko (*Klebsiella oxytoca*), Kp (*K. pneumonia*), Pa' (*Pantoea ananatis*), Pa (*Pectobacterium atrosepticum*), Pw (*P. wasabiae*), Se Ana (*Salmonella enterica* subsp. enterica serovar Anatum), Se Ari (*S. enterica* subsp. arizonae serovar 62:z4,z23:-), Se Dia (*S. enterica* subsp. diarizonae serovar 60:r:e,n,x,z15), Se Hvi (*S. enterica* enterica subsp. enterica serovar Hvittingfoss), Se Ind (*S. enterica* subsp. enterica serovar Indiana), Se Mue (*S. enterica* subsp. enterica serovar Muenster), Se Nch (*S. enterica* subsp. enterica serovar Nchanga), Se Slo (*S. enterica* subsp. enterica serovar Sloterdijk), and Yr (*Yersinia rohdei*). An asterix following the strain name indicates strains containing the LEE. Residues of potential importance for H-NST function are indicated in color or bold, where the Ala16 residue previously reported to be important for H-NST activity is shown in blue, the Arg60 and Arg63 residues that could be involved in DNA-binding by H-NST are shown in red and green respectively. The conserved arginine residue shown in red is involved in DNA-binding of H-NS and Ler, while the arginine in green present in Ler and H-NST is involved in DNA-binding by Ler [36]. An asterisk represents identical amino acids, a colon represents a conserved amino acid substitution and a dot indicates a semi-conserved amino acid substitution. The heptadic repeat defined for H-NST_EPEC_ is indicated above the sequence alignment. The letters *a* and *d* represent hydrophobic residues, *e* and *g* represent charged residues, whereas positions *b* and *c* can be occupied by any residue in the repeat. (B) Sequence alignment of the C-terminal regions of Ler, H-NS and H-NST generated by ClustalW. The boxed region indicates the conserved DNA-binding motif for the H-NS family of proteins. The annotation used for the alignment is as described for panel A.

Since H-NST from EPEC E2348/69 positively affects the LEE we carried out BLAST searches to determine the presence of H-NST among A/E pathogens and other *Enterobacteriaceae* encoding H-NS as described in *Material and Methods*. We identified H-NST from 65 *Enterobacteriaceae* strains of which 32% contained the LEE (figure 2A). Those strains included 14 EPEC strains, *C. rodentium* and six LEE-positive, *bfp-* and *stx-* strains of unclassified phylogenetic lineage [44], which were serotypes O157:H45 (strains C844-97, C639-08 and 3003), O114:H49 (C262-10), O157:H39 (TW07793) and O157:H- (C93-11). H-NST homologs were not found in any Shiga toxin-producing strains such as EHEC O157:H7 and *Shigella* sp., which could be due to the fact that other pathogenicity islands occupy the *asnW* and *serU* loci of the H-NST-encoding P4-like phage in these strains as is the case of the CP933U island in EDL933 and *Shigella flexneri*. H-NST is also present in other *E. coli* pathotypes including numerous UPEC strains, enteroaggregative *E. coli* 042 and adherent invasive *E. coli* (CUMT8), the latter which has been associated with Crohn's disease [51]. We further identified H-NST in commensal *E. coli* including some reference strains from the Human Microbiome Project (NIH). Interestingly, H-NST also is conserved in other human pathogens such as *Klebsiella pneumonia* and *K. oxytoca* as well as in *Salmonella enterica*, where H-NST is present in three subspecies and eight serovars that are associated with food poisoning outbreaks worldwide [52]. Further, H-NST is present in phytopathogenic bacteria targeting potato and rice plants including *Dickeya zeae*, *P. atrosepticum*, *Pectobacterium wasabiae* and *Pantoea ananatis*, the latter of which is an opportunistic human pathogen causing bacteremia [53]. H-NST is present in some A/E pathogens, in other human pathogens such as *Salmonella* and in plant pathogens that utilize H-NS-mediated regulation of virulence factors. This widespread distribution suggests a global regulatory effect of H-NST, prompting us to further investigate the molecular basis for H-NST function on the regulation of *LEE* expression.

### Identification of residues important for H-NST-mediated induction of *LEE*-encoded proteins and A/E lesion formation

The current literature suggests that H-NST, like Ler, functions by negatively affecting H-NS-mediated repression of gene expression probably by displacing H-NS bound to regulatory DNA sites [37]. However, it remains to be determined whether H-NST, like Ler and H-NS, binds to DNA. To gain further insight into the molecular mechanism by which H-NST regulates gene expression, we identified residues of potential functional interest based on sequence alignment of H-NST, Ler and H-NS. We used ClustalW2 [45], [46] to align the C-terminal regions of Ler and H-NS that contain the DNA-binding motif with the C-terminal half of H-NST, since only the first 40 residues of H-NST share a high similarity with the N-terminus of H-NS. The alignment revealed that the conserved DNA-binding motif of the H-NS family of proteins including H-NS and Ler is absent from H-NST (figure 2B). Interestingly, despite the absence of the DNA-binding motif present in the H-NS family of proteins, H-NST contains an arginine residue at position 60 that aligned with the Ler Arg90 and H-NS Arg114, which are essential for DNA-binding activity of those regulators [36], [54], [55]. Also, the Ler Arg93 residue that is associated with stabilization of the Ler-*LEE* DNA complex, and was suggested to play a role in Ler regulation of *LEE* expression [36], is conserved in H-NST as Arg63 (figure 2B). Indeed, H-NST residues Ala16 and Arg60 are conserved as indicated in the multiple sequence alignment (figure 2A). To assess the role of Arg60 and Arg63, which potentially could be part of a DNA-binding domain, we performed site-directed mutagenesis on pQEH-NST to substitute the arginine residues with glutamine resulting in plasmids encoding the H-NST mutants R60Q and R60Q/R63Q as described in *Materials and Methods*.

The H-NST Ala16 residue was previously identified as essential for H-NST function based on the finding that a H-NST A16V mutant was incapable of regulating *proU* expression and that H-NST from UPEC containing valine at position 16 is nonfunctional [37]. The authors speculated that the loss of activity was caused by the inability of the H-NST A16V mutant to engage in higher order oligomeric protein-protein interactions with H-NS. To determine whether H-NST Ala16 also is important for the H-NST-mediated regulation of *LEE* expression and the A/E lesion phenotype, we constructed the H-NST A16V mutant using site-directed mutagenesis of pQEH-NST. Evaluation of the sequence around Ala16 revealed a potential heptadic repeat within the coiled-coil element of H-NST of which Ala16 is the first residue (figure 2A). A canonical heptadic repeat is a seven amino acid residue long sequence that forms coiled-coil secondary structures, which are involved in protein-protein interactions [56]. The proposed repeat in H-NST contains the characteristics of a classic heptadic repeat since positions *a* and *d* occupy hydrophobic residues, positions *e* and *g* commonly contain charged residues, whereas positions *b* and *c* are random residues in the repeat [56], [57] (figure 2A). Interestingly, the H-NST heptadic repeat is composed of alanines and leucines occupying the *a* and *d* positions, whose side chains are less bulky compared to that of isoleucine and valine. Therefore, to assess the importance for oligomerization of having an alanine residue at position 16, we substituted Ala16 with Leu resulting in the mutant H-NST A16L, which potentially could restore H-NST function since the leucine does not harbor a β-branched chain, compared to that of valine and isoleucine.

**Figure 3:**
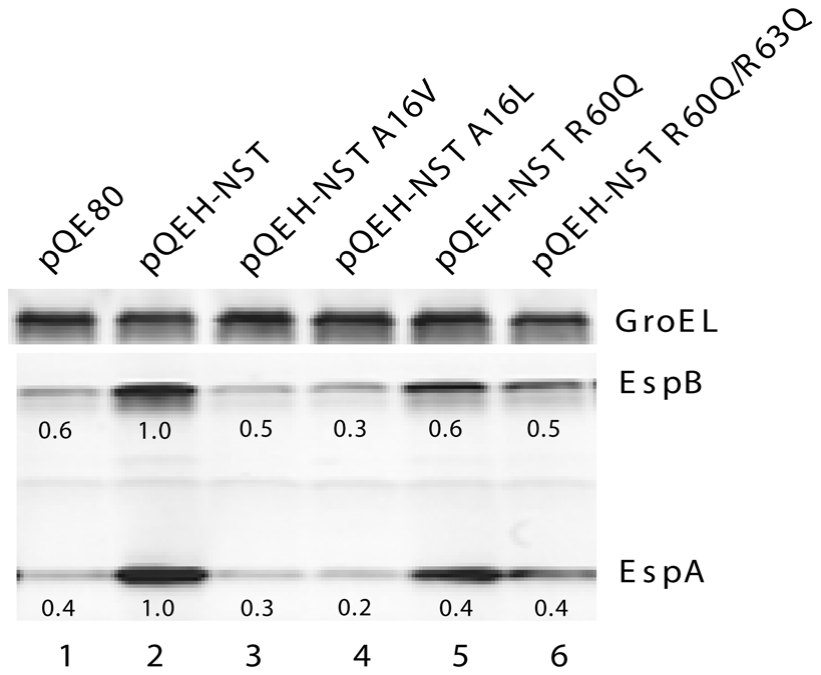
H-NST mutants exhibit decreased ability to induce the production of *LEE*-encoded proteins. The effect of wild type H-NST and mutant derivatives on *LEE*-encoded protein levels was determined by western analysis as described in *Materials and Methods*. EHEC strain TUV93-0 containing the empty vector pQE80 (lane 1), TUV93-0 containing constructs producing wild type EPEC H-NST (lane 2) or the H-NST mutant derivatives H-NST A16V (lane 3), H-NST A16L (lane 4), H-NST R60Q (lane 5) and HNST R60Q/R63Q (lane 6) were grown in LB to a density of OD_600_∼0.5 and *hnsT* expression was induced by 0.5 mM IPTG for 60 min. Levels of EspA, EspB and GroEL were detected in total protein by western analysis using polyclonal antisera against the respective proteins as indicated. GroEL served a loading control for total protein. The relative levels of EspA and EspB normalized to that of GroEL are indicated by numbers below the protein bands. Data shown are representative of four independent experiments.

We tested the ability of H-NST mutants A16V, A16L, R60Q and R60Q/R63Q to affect levels of *LEE*-encoded proteins of EHEC in the exponential phase of growth. Results revealed a decreased ability of all H-NST mutants to induce the production of EspA and EspB compared to wild type H-NST (figure 3, lanes 3–6). Specifically, the H-NST mutants A16V and A16L exhibited decreased ability to induce *LEE* expression as reflected by a 2- to 6-fold decrease in EspA and EspB levels relative to wild type H-NST (figure 3, lanes 3–4). H-NST mutants at residues Arg60 and Arg63 also showed a decreased ability to affect *LEE*-encoded protein levels compared to the wild type H-NST with a 2- to 3-fold decrease in EspA and EspB levels (figure 3, lanes 5–6).

**Figure 4:**
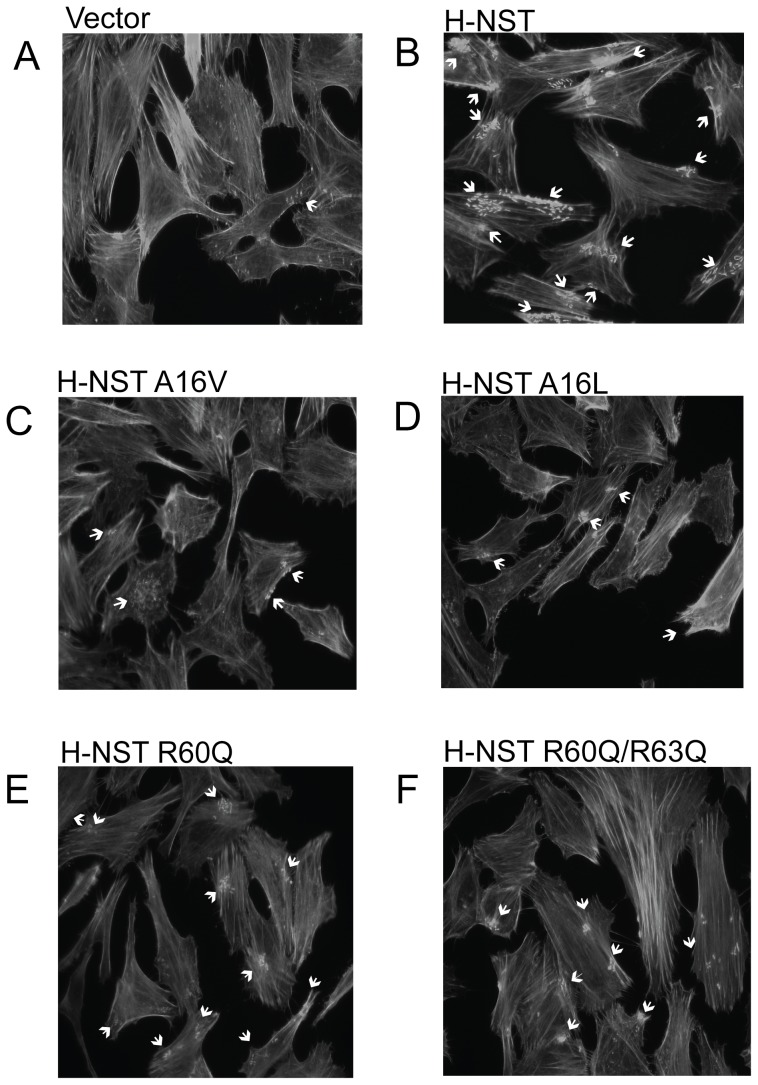
H-NST induces A/E formation. FAS assays were used to determine the effect of H-NST on A/E lesion formation of EHEC as described in *Materials and Methods*. HeLa cell monolayers were co-cultured for four hours with EHEC strain TUV93-0 containing the empty vector pQE80 (A), constructs producing wild type EPEC H-NST (B) or the H-NST mutant derivatives H-NST A16V (C), H-NST A16L (D), H-NST R60Q (E) and HNST R60Q/R63Q (F). The images of FITC phalloidin-stained actin of infected HeLa cells are representative of three independent experiments. Arrows indicate examples of A/E lesions.

To determine whether the decreased ability of the H-NST mutants to induce accumulation of *LEE*-encoded proteins compared to wild type H-NST affected T3SS function, we evaluated the A/E lesion phenotype of EHEC producing wild type and H-NST mutant derivatives using the fluorescent actin staining assay (FAS). Actin filaments are stained by FITC-phalloidin in this assay to visualize condensed actin indicative of A/E lesions. FAS assays for EHEC typically involve a 5–6 h incubation time [48], [58]. We infected HeLa cells with EHEC producing wild type or mutant H-NST for 4 h, a point at which A/E lesion formation is minimal yet detectable [48]. Infection with EHEC containing the vector control demonstrated minimal actin pedestal formation as is expected with the co-infection time of 4 h (figure 4A). In contrast, EHEC producing wild type H-NST showed a high degree of A/E lesion formation (figure 4B), which correlates with increased EspA and EspB levels (figure 1B). H-NST mutants A16V and A16L both showed reduced A/E lesion formation compared to wild type H-NST (figure 4, panels C–D), indicating an important role of the Ala16 residue for H-NST to induce this virulence phenotype in EHEC. The H-NST R60Q and R60Q/R63Q mutants showed a slightly reduced degree of A/E lesion formation compared to wild type H-NST with the double arginine mutant showing a larger reduction than the single arginine mutant (figure 4, panels E–F). Neither of the arginine mutants exhibited impaired function to the extent seen with the H-NST A16V and A16L mutants, which correlates with the effect of these respective mutants on EspA and EspB levels (figure 3). Altogether, the H-NST Ala16 mutant exhibited the most severe effect on the ability of H-NST to affect the level of *LEE*-encoded proteins and induce pedestal formation, further supporting the functional importance of the Ala16 residue. The decreased activity of H-NST mutant at the Arg60 and Arg63 residues suggest functional importance of these residues, which potentially could be involved in DNA-binding.

### H-NST binds to the regulatory regions of *LEE1* and *LEE3*

**Figure 5:**
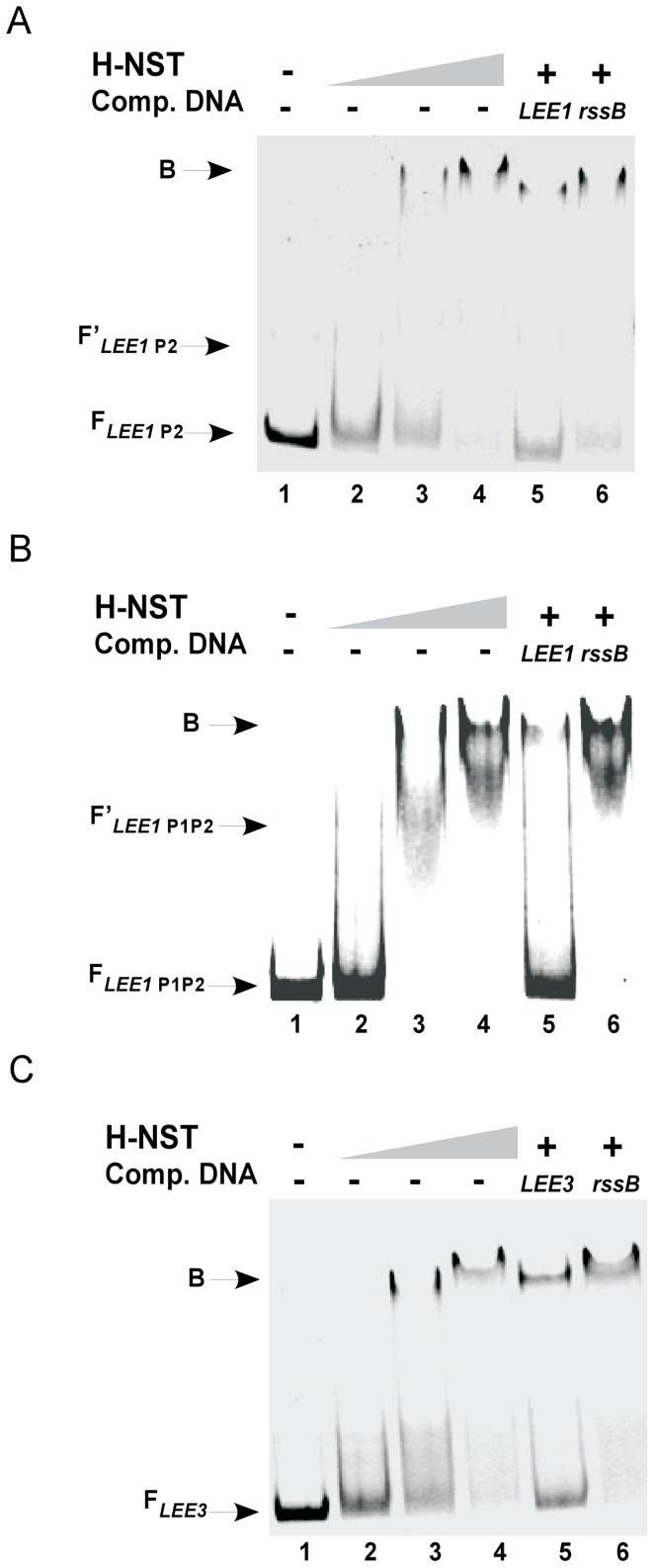
H-NST binds to regulatory regions of *LEE1* and *LEE3*. The binding of H-NST to fluorescently-labeled *LEE* DNA targets was determined using electrophoretic mobility shift assays as described in *Materials and Methods*. Fluorescently-labeled DNA fragments containing the proximal promoter region of *LEE1* (*LEE1* P2) (A), the distal and proximal promoter regions of *LEE1* (*LEE1* P1P2) (B), and a regulatory region of *LEE3* (*LEE3*) (C) were incubated in the absence of H-NST (lane 1) and with increasing concentrations of H-NST (lane 2: 25 nM; lane 3: 50 nM; and lane 4: 100 nM). To determine the binding specificity of H-NST, fluorescently-labeled *LEE* DNA targets were incubated with 100 nM H-NST in the presence of unlabeled competitor DNA fragments (Comp. DNA) containing specific (*LEE1* P2, *LEE1* P1P2 or *LEE3*) (lane 5) or non-specific (*rssB)* (lane 6) DNA targets in the ratios 1∶6 and 1∶12 respectively. Bound and unbound DNA fragments were separated by PAGE on a 4–20% TBE gel. Arrows labeled F indicate unbound DNA, while F′ arrows indicate an unbound DNA subpopulation. The arrows labeled B indicate DNA fragments with H-NST bound. Data shown are representative of three independent experiments.

H-NS and Ler both modulate *LEE* operon expression through binding to DNA. The *LEE1*-encoded *ler* is expressed from two promoters named the distal (P1)- and proximal (P2) promoters with the distal promoter being the major promoter for *LEE1* expression [59]. The molecular mechanism of Ler and H-NS-mediated regulation of the *LEE1* and *LEE2/LEE3* operons has been well studied [10], [30]. Specifically the curvature of DNA and the oligomeric state of the regulator is essential for the ability of these H-NS family proteins to bind *LEE* DNA [32], [36], [60]. We analyzed the ability of H-NST to bind to the regulatory regions of *LEE1* and *LEE3* DNA targets contained in EHEC using electrophoretic mobility shift assays (EMSA) (figure 5). We purified a C-terminal hexahistidine-tagged H-NST protein to about 95% purity and determined the ability of H-NST to bind to fluorescently-labeled *LEE1* and *LEE3* DNA fragments. We demonstrated that increasing concentrations of H-NST shifted the *LEE1* P2 DNA fragment, which contains the proximal *LEE1* P2 promoter and this 10 bp sequence, whereas the mobility of the DNA fragment in the absence of H-NST was unchanged (figure 5A, lanes 2–4). The DNA-binding specificity of H-NST to *LEE1* P2 DNA was tested by adding unlabeled specific *LEE1* P2 DNA and nonspecific DNA encoding *rssB* in the respective ratios of 6∶1 and 12∶1 along with the labeled DNA target. The binding of H-NST to the labeled *LEE1* P2 DNA fragment was outcompeted in the presence of the unlabeled specific probe as reflected by a partial downshift of the labeled *LEE1* P2 DNA fragment, whereas the DNA fragment shifted by H-NST remained unchanged in the presence of unlabeled *rssB* DNA (figure 5A, lanes 5 and 6), indicating that binding of H-NST to *LEE1* P2 DNA is specific. The DNA sequence amplified from EHEC contained in the *LEE1* P2 DNA fragment shares 97% identity to that in EPEC *LEE1* P2 promoter [59]. We tested whether H-NST binds to the *LEE1* P2 DNA fragment from EPEC, and as expected H-NST also bound to the *LEE1* regulatory region from EPEC (unpublished data).

To gain insight into the binding of H-NST to the complete regulatory region of *LEE1* containing both P1 and P2 promoters, we evaluated the binding of H-NST to the *LEE1* P1P2 DNA target. H-NST bound specifically to *LEE1* P1P2 DNA as demonstrated by a downshift of the bound labeled fragment in the presence of specific unlabeled *LEE1* P1P2 DNA as opposed to the presence of unlabeled nonspecific *rrsB* DNA (figure 5B, lanes 5–6). The presence of increasing H-NST concentrations caused a greater degree of shift for the *LEE1* P1P2 DNA fragment compared to that of the *LEE1* P1 DNA fragment, suggesting that the binding affinity of H-NST for the *LEE1* P1P2 DNA fragment harboring the entire *LEE1* regulatory region might be higher than that of the *LEE1* P1 fragment that contains only parts of the *LEE1* regulatory region (figure 5A–B, lanes 3–4). Since Ler regulation of the *LEE2/LEE3* is well characterized and that the molecular mechanism of Ler binding to the *LEE3* regulatory region recently was elucidated [30], [36], we wanted to determine whether H-NST binds to this region. Indeed, H-NST bound at increasing concentrations to the *LEE3* DNA fragment containing the *LEE3* regulatory region containing a 10 bp Ler target sequence identified by Codiero *et al* 2011 [36] (figure 5C, lanes 2–4). As for the *LEE1* regulatory region, H-NST exhibited specific binding to the *LEE3* DNA fragment as reflected by a partial downshift of the labeled *LEE3* DNA fragment with H-NST bound in the presence of the specific unlabeled *LEE3* DNA fragment, whereas non-specific unlabeled *rrsB* DNA had no effect on the *LEE3* DNA fragment shifted by H-NST (figure 5C, lanes 5–6). Altogether, we demonstrated that H-NST binds in a specific manner to the regulatory regions of *LEE1* and *LEE3*, which to our knowledge represents the first demonstration that H-NST binds to DNA.

### The H-NST Arg60 and Arg63 residues of the C-terminal region are important for DNA-binding

**Figure 6:**
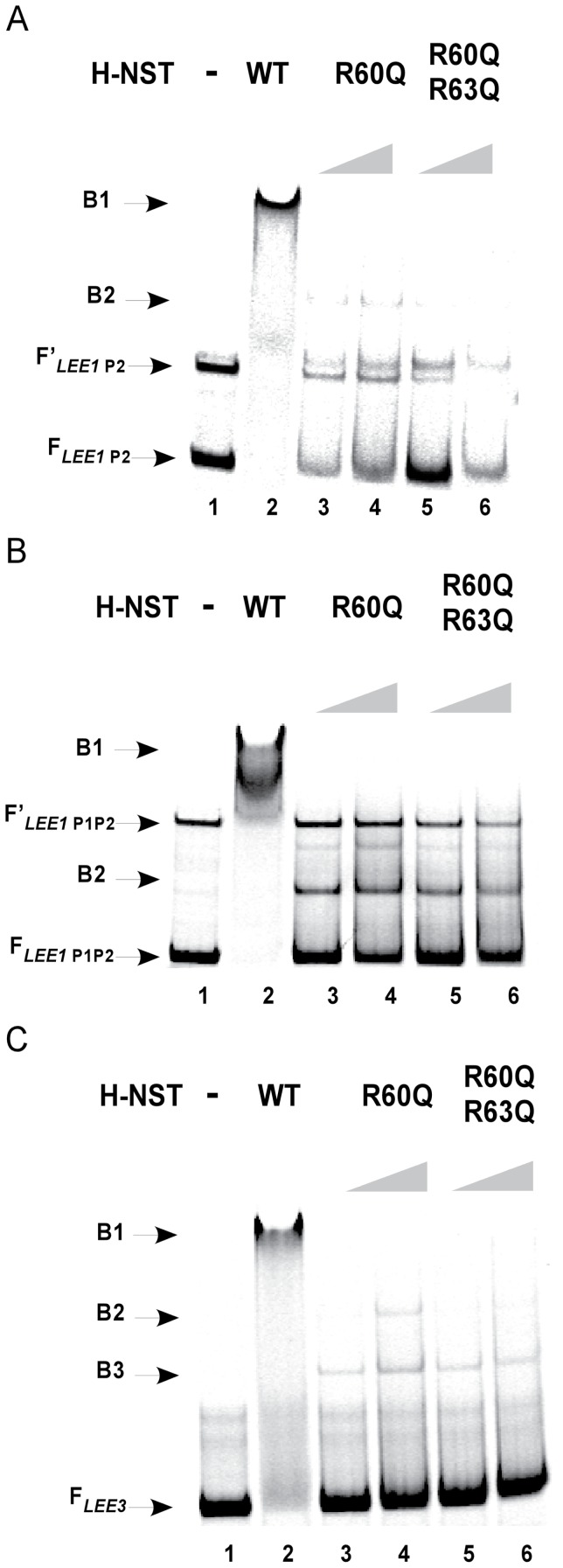
The H-NST C-terminal Arg60 and Arg63 residues positively affect DNA-binding. Electrophoretic mobility shift assays were used to assess the binding of wild type H-NST and H-NST mutants containing the substitutions R60Q and R60Q/R63Q to the fluorescently-labeled *LEE* DNA targets: *LEE1* P2 (A), *LEE1* P1P2 (B) and *LEE3* (C). The DNA fragments were incubated in the absence of H-NST (lane 1), with 100 nM wild type H-NST (lane 2), H-NST R60Q (lane 3: 50 nM and lane 4: 100 nM), and with H-NST R60Q/R63Q (lane 5: 50 nM and lane 6: 100 nM). Arrows labeled F indicate unbound DNA, while F′ arrows indicate an unbound DNA subpopulation. The arrows labeled B1 indicate fully shifted DNA fragments, while B2 and B3 indicate partially shifted DNA fragments. Data shown are representative of two independent experiments.

We demonstrated that the single mutant H-NST R60Q and the double mutant H-NST R60Q/R63Q exhibit a decreased ability to positively affect the levels of *LEE-*encoded proteins and induce A/E lesion formation (figures 3 and 4E–F). This finding highlights the functional importance of these arginine residues located in the C-terminal region of H-NST. Since H-NST residues Arg60 and Arg63 aligned with arginine residues of Ler known to be important for DNA-binding (figure 2B), we tested whether these residues played a role in DNA-binding by H-NST. To this end, we purified H-NST mutants R60Q and R60Q/R63Q to determine their ability to bind *LEE* DNA (figure 6). The H-NST R60Q and R60Q/R63Q mutants both showed diminished ability to bind to the *LEE1* P2, *LEE1* P1P2 and *LEE3* DNA targets compared to wild type H-NST (figure 6A–C, lanes 3–6), indicating an importance of these arginines in DNA-binding by H-NST. Further, the H-NST R60Q/R63Q double mutant was less capable of binding the *LEE1* P2 and *LEE3* DNA fragments as reflected by decreased amounts of the complex designated as B2 when increasing concentrations of the H-NST double mutant rather than the R60Q single mutant were present (figure 6A and 6C, compare lanes 5–6 with lanes 3–4), suggesting that H-NST Arg63 like the corresponding Ler Arg93 residue positively affects DNA-binding. Notably, the H-NST R60Q and R60Q/R63Q mutants did not exhibit a differential effect on binding to the *LEE1* P1P2 fragment (figure 6B, lanes 3–6), which could be due to the possibility that H-NST binds more strongly to the *LEE1* P1P2 DNA fragment than to the shorter *LEE1* P2 DNA fragment. The EMSA analyses involving H-NST mutated at the Arg60 and Arg63 residues correlated with the decreased ability of the mutants to induce levels of *LEE*-encoded proteins and A/E lesion formation, and revealed an important role of these arginine residues in DNA-binding by H-NST.

### H-NST Ala16 is important for DNA-binding

**Figure 7:**
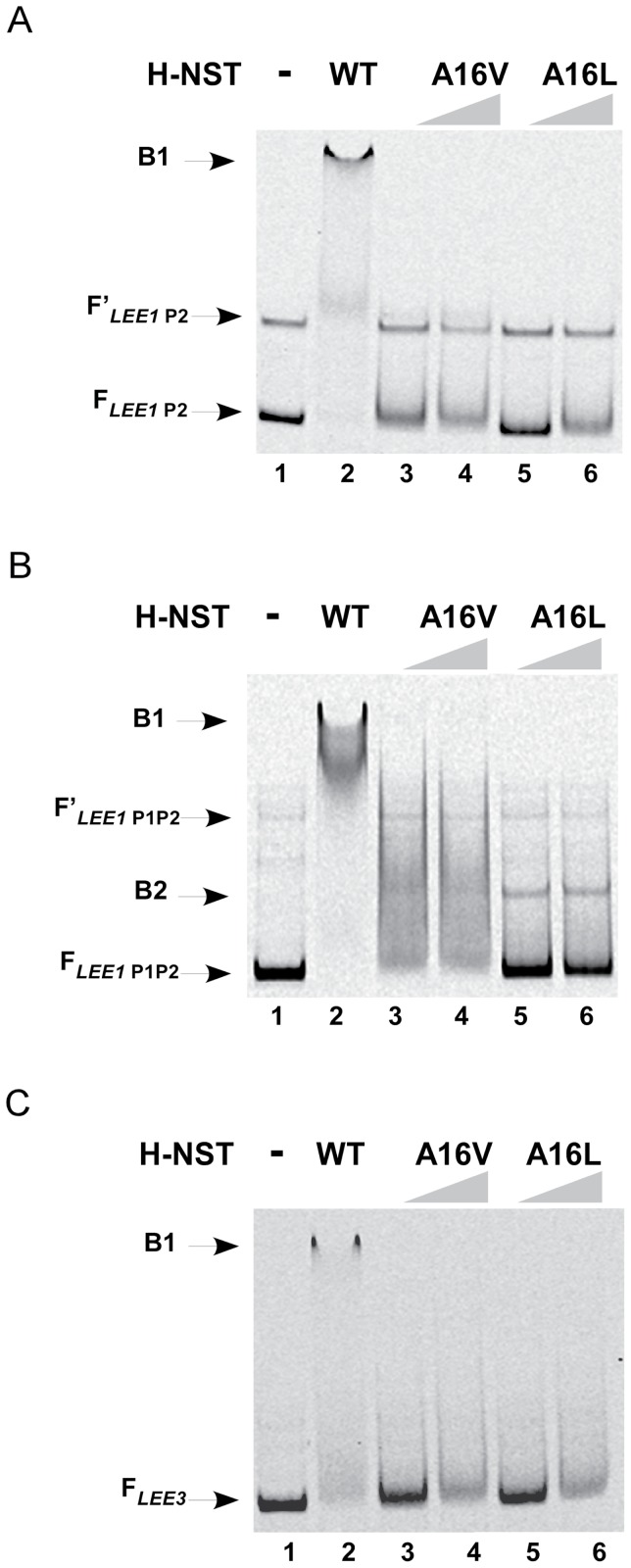
The H-NST Ala16 residue is important for DNA-binding. The binding of wild type H-NST and mutant H-NST to *LEE* DNA targets was determined by electrophoretic mobility shift assays. Fluorescently-labeled DNA fragments containing *LEE1* P2 (A), *LEE1* P1P2 (B) and *LEE3* (C) regulatory regions were incubated in the absence of H-NST (lane 1), with 100 nM H-NST (lane 2); and with the H-NST mutants H-NST A16V (lane 3: 50 nM and lane 4: 100 nM H-NST) and H-NST A16L (lane 5: 50 nM and lane 6: 100 nM). Bound and unbound DNA fragments were resolved by PAGE on a 4–20% TBE gel. Arrows labeled F indicate unbound DNA, while F′ arrows indicate an unbound DNA subpopulation. The arrows labeled B1 indicate fully shifted DNA fragments, while B2 indicates partially shifted DNA fragments. Data shown are representative of three independent experiments.

We demonstrated that the H-NST Ala16 residue that is required for H-NST oligomerization with H-NS [37] is also required for H-NST to cause an increase in levels of *LEE*-encoded proteins and induce pedestal formation on epithelial cell monolayers (figures 3 and 4C–D). In addition to playing a role in oligomerization, we determined whether Ala16 is also involved in DNA-binding by H-NST. We purified the H-NST mutants A16V and A16L and tested their ability to bind *LEE* DNA targets (figure 7). EMSA analyses revealed that neither of these H-NST mutants bound to *LEE1* P2 and *LEE3* DNA fragments as reflected by the lack of a change in mobility of these DNA fragments (figure 7A and C, lanes 3–4). Nevertheless, the H-NST A16V and A16L mutants exhibited weak binding to the *LEE1* P1P2 DNA as reflected by the appearance of a defined weak partially shifted band, which in particular appeared in the presence of the H-NST A16L mutant (indicated as B2 in figure 7B, compare lanes 3–4 and 4–5 with lane 1). The smeared appearance of the *LEE1* P1P2 DNA fragment incubated with the H-NST A16V mutant could reflect a weak and/or indiscriminant binding to this DNA fragment containing the complete *LEE1* regulatory region (figure 7B, lanes 3–4). In all, our results indicate that the H-NST Ala16 residue is important for DNA-binding activity of H-NST. The importance of having the conserved Ala16 residue for H-NST function was demonstrated by the inability of the H-NST A16L mutant to restore H-NST function. It remains to be demonstrated whether oligomerization of H-NST is a prerequisite for DNA-binding or if the Ala16 residue directly affects the binding of H-NST to DNA, an issue which is beyond the scope of this study.

### H-NST positively affects the binding of Ler to *LEE3* DNA pre-bound by H-NS

**Figure 8:**
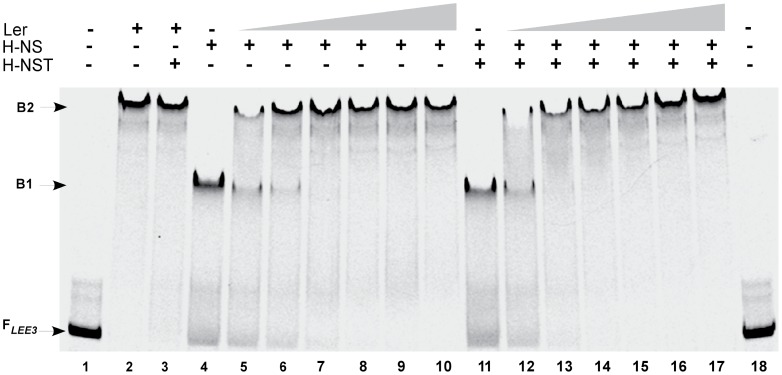
H-NST promotes the binding of Ler to *LEE3* DNA bound by H-NS. Fluorescently-labeled DNA fragments containing the *LEE3* regulatory region were incubated alone (lanes 1 and 18), with 175 nM Ler (lane 2), with 175 nM Ler and 50 nM H-NST (lane 3), with 50 nM H-NS (lane 4), with 50 nM HNS in the presence of increasing Ler concentrations (50, 100, 125, 150, 175 and 250 nM Ler; lanes 5–10 respectively), with 50 nM H-NST and 50 nM H-NS (lane 11), with 50 nM HNS and 50 nM H-NST along with increasing Ler concentrations (50, 100, 125, 150, 175 and 250 nM Ler; lanes 12–17). Bound and unbound DNA fragments were separated by PAGE on a 4–20% TBE gel. The arrow labeled F indicates unbound DNA. DNA fragments shifted by H-NS and/or H-NST are indicated as B1, while DNA fragments shifted by Ler are labeled B2. Data shown are representative of three independent experiments.

The present mechanism for H-NST function suggests that by forming dominant-negative-acting oligomers with H-NS, H-NST prevents H-NS from modulating H-NS regulon expression [37]. We demonstrated that H-NST functions independently of *ler* (figure 1B). To obtain additional insight into the role of H-NST-mediated regulation of the *LEE*, we used EMSA analyses to determine whether H-NST promotes the binding of Ler to *LEE* DNA that already has H-NS bound. To allow protein-protein interactions to occur we added H-NST and/or H-NS prior to incubation with the *LEE3* DNA target, and then added increasing amounts of Ler to determine whether the presence of H-NST helped binding of Ler to *LEE3* DNA pre-bound by H-NS. Binding of H-NST and/or H-NS to *LEE3* DNA was visualized as a shifted band (indicated as B1), which migrated at the same position regardless of whether one or both proteins were present (figure 8, lanes 4 and 11). The presence of H-NST did not affect Ler binding to the *LEE3* DNA fragment in the absence of H-NS as reflected by the similar shift observed (indicated as B2 in figure 8, lanes 2–3). Ler bound at increasing concentrations to *LEE3* DNA pre-bound with either H-NS (figure 8, lanes 5–10) or both H-NS and H-NST (figure 8, lanes 12–17) resulted in the shifted band indicated B2. Interestingly, Ler at a 100 nM concentration caused a complete shift of DNA pre-bound to H-NS (indicated as B1) only in the presence of H-NST (figure 8, compare lanes 6 and 13). This result suggests that H-NST could help Ler outcompete H-NS bound to *LEE* DNA, which correlates with our finding that H-NST positively affects the LEE.

## Discussion

The expression of virulence factors including those of A/E pathogens encoded in the *LEE* are subject to extensive regulation involving many environmental signals to ensure that virulence-associated factors are produced under conditions optimal for infection. Under such conditions, silencing of virulence gene expression by the global modulator H-NS is counteracted by the major activator of virulence gene expression in A/E pathogens, Ler. H-NST present in EPEC, which is defined as a truncated H-NS derivative lacking the DNA-binding domain, was previously shown to positively affect the relief of H-NS-mediated repression in *E. coli* K-12 by interacting with H-NS to prevent H-NS oligomerization [37]. Since H-NST could provide an additional mechanism that promotes the relief from H-NS-mediated repression of virulence gene expression in A/E pathogens and in other pathogens encoding H-NST (figure 2A), we evaluated the role of H-NST on the production of the virulence-associated *LEE*-encoded factors. To determine whether H-NST is required for the production of *LEE*-encoded proteins in EPEC we deleted *hnsT* and tested the effect on EspA and EspB levels, which appeared unaffected in the absence of H-NST (unpublished data), suggesting that H-NST is dispensable for *LEE* expression under the growth in DMEM. However, H-NST when expressed from an inducible promoter positively affected *LEE*-encoded protein levels and subsequently A/E lesion formation of EHEC (figures 3 and 4A–B), indicating that H-NST can positively impact the expression of virulence genes in response to a yet-to-be identified environmental signal(s).

Arginine residues are commonly involved in DNA-binding by proteins [61] including those of the H-NS family such as Ler. Here, we demonstrated that the arginine residues important for DNA-binding by H-NS (Arg114) and Ler (Arg90 and Arg93) are present in H-NST as Arg60 and Arg63 (figure 2B). This observation propelled us to explore the DNA-binding potential of H-NST by testing the ability of H-NST to bind to *LEE* target DNA *in vitro*. We demonstrated that H-NST binds to DNA fragments containing the regulatory regions of *LEE1* and *LEE3* (figure 5), which is the first demonstration of DNA-binding by H-NST. Further, our data revealed that residues Arg60 and Arg63 are important for H-NST DNA-binding activity, which correlates with a positive effect of these residues on the induction of *LEE*-encoded protein levels and A/E lesion formation (figures 3 and 4E–F). Though DNA-binding by H-NST R60Q and R60Q/R63Q mutants was diminished compared to that of wild type H-NST, these mutants still exhibited residual DNA-binding (figure 6), suggesting that the positively charged nature of the H-NST C-terminal region, due to the high prevalence of lysine and arginine residues, positively influences H-NST DNA-binding in addition to the functionally important Arg60 and Arg63 residues. Altogether, we demonstrate that H-NST binds to DNA and that this DNA-binding activity is required for H-NST to affect the expression of the *LEE* contained in A/E pathogens.

We demonstrated that the Ala16 residue, which is known to be functionally important for H-NST to positively affect the expression of the H-NS-repressed genes *proU* and *bgl* in *E. coli* K-12 [37], is also required for H-NST to control the *LEE* and A/E lesion formation (figures 3 and 4C-D), further indicating that H-NST affects the expression of horizontally-acquired virulence-associated genes. H-NST Ala16 was reported to be functionally important for H-NST to counteract H-NS-mediated repression based on the finding that a H-NST A16V mutant exhibited a diminished ability to derepress *proU* expression, which was suggested to be due to the inability of the A16V mutant to form functional oligomers [37]. The Ala16 residue located in the proposed second α-helix of the N-terminal coiled-coil region of the oligomerization domain occupies the first position in the predicted heptadic repeat (figure 2A). In case of the inactive H-NST A16V mutant, having a valine at position 16 as the first residue in the heptadic repeat could be associated with steric constraints since valine harbors a β-branched side-chain absent from alanine, which could negatively affect coiled-coil packing [62]–[64], and thereby could prevent oligomerization of H-NST itself and with H-NS. We therefore expected that the introduction of leucine that contains an unbranched β chain at position 16 in H-NST would result in a functional H-NST mutant. However, our data revealed that a H-NST A16L mutant like the A16V mutant was incapable of inducing *LEE*-encoded protein levels and A/E lesion formation (figure 3, 4C–D), suggesting that the presence of a residue containing a short β chain at position 16 is required for H-NST functionality. We tested the DNA-binding capacity of H-NST mutants A16V and A16L, and found that these mutants exhibited diminished DNA-binding activity (figure 7), suggesting that the ability to oligomerize could be important for H-NST to bind DNA. However, we cannot exclude the possibility that Ala16 also directly affects DNA-binding by H-NST.

Interestingly, our data indicated that H-NST positively affects the binding of Ler to the *LEE3* regulatory region pre-bound by H-NS (figure 8), suggesting that H-NST helps Ler-binding to DNA perhaps by promoting the dissociation of H-NS from the *LEE3* regulatory region. It is possible that H-NST when bound to DNA can change DNA topology to a conformation that is more suitable for DNA-binding by Ler than H-NS. The finding that H-NST affects *LEE* expression independently of *ler* further supports a model in which H-NST positively regulates expression through H-NS. Whether H-NST does so by modulating the DNA curvature by binding to DNA and/or prevents the binding of H-NS to DNA by forming H-NST/H-NS oligomers as previously suggested [37], [41], is unresolved. Further investigation, beyond the scope of this current study, is required to elucidate the molecular basis for the H-NST function, in particular with regard to how H-NST promotes the binding of Ler to DNA pre-bound by H-NS.
